# NF-YA Overexpression in Lung Cancer: LUAD

**DOI:** 10.3390/genes11020198

**Published:** 2020-02-14

**Authors:** Eugenia Bezzecchi, Mirko Ronzio, Valentina Semeghini, Valentina Andrioletti, Roberto Mantovani, Diletta Dolfini

**Affiliations:** 1 Dipartimento di Bioscienze, Università degli Studi di Milano, Via Celoria 26, 20133 Milano, Italy; 2 Internal Medicine VIII, University Hospital Tübingen. Otfried-Müller-Str. 14, 72076 Tübingen, Germany

**Keywords:** lung cancer, LUAD, transcription factors, TCGA, CCAAT box, NF-YA, alternative splicings

## Abstract

The trimeric transcription factor (TF) NF-Y regulates the CCAAT box, a DNA element enriched in promoters of genes overexpressed in many types of cancer. The regulatory NF-YA is present in two major isoforms, NF-YAl (“long”) and NF-YAs (“short”). There is growing indication that NF-YA levels are increased in tumors. Here, we report interrogation of RNA-Seq TCGA (The Cancer Genome Atlas)—all 576 samples—and GEO (Gene Expression Ominibus) datasets of lung adenocarcinoma (LUAD). NF-YAs is overexpressed in the three subtypes, proliferative, inflammatory, and TRU (terminal respiratory unit). CCAAT is enriched in promoters of tumor differently expressed genes (DEG) and in the proliferative/inflammatory intersection, matching with KEGG (Kyoto Encyclopedia of Genes and Genomes) terms *cell-cycle* and *signaling*. Increasing levels of NF-YAs are observed from low to high CpG island methylator phenotypes (CIMP). We identified 166 genes overexpressed in LUAD cell lines with low NF-YAs/NF-YAl ratios: applying this centroid to TCGA samples faithfully predicted tumors’ isoform ratio. This signature lacks CCAAT in promoters. Finally, progression-free intervals and hazard ratios concurred with the worst prognosis of patients with either a low or high NF-YAs/NF-YAl ratio. In conclusion, global overexpression of NF-YAs is documented in LUAD and is associated with aggressive tumor behavior; however, a similar prognosis is recorded in tumors with high levels of NF-YAl and overexpressed CCAAT-less genes.

## 1. Introduction

Lung cancer is the second leading cause of death in males of industrialized countries, and is a rising concern in females. There are several types of lung cancer, with the vast majority—80% to 90%—belonging to non-small cell lung carcinoma (NSCLC) [1]. Genetic and molecular characterization of NSCLC tumors has clearly separated two fundamentally distinct types, lung squamous cell carcinoma (LUSC) and lung adenocarcinoma (LUAD). LUSC and LUAD were further subclassified in four and three sub-types, respectively, via gene expression analysis and identification of signatures [2,3]. Changes in transcriptomes upon cellular transformation are a consequence of genetic mutations and/or epigenetic changes, which impact on transcription rates. In turn, these are ultimately caused by the levels or usage of transcription factors (TFs) and chromatin modifying cofactors [4]. Alteration in the protein structure or in the expression of specific TFs are causative of tumorigenic changes in gene expression. 

Gene expression analysis through microarray profilings found “signature” genes in many cancer types; in addition to serving the scope of investigating mechanistically the role of single genes, these studies were used to systematically identify TFBSs (transcription factor binding sites) in promoters of genes overexpressed in cancers. This exercise has often found the CCAAT box as specifically enriched [5,6,7,8,9,10]. CCAAT is a well characterized and important DNA element of promoters and enhancers; in promoters, it is located at a −60/−100 location from the TSS, and it is important for high-level expression of the targeted genes [11]. NF-Y is the TF acting on the CCAAT box. It is formed by the sequence-specific NF-YA and the histone fold domain (HFD) dimer NF-YB/NF-YC [5]. NF-YA is alternatively spliced at the N-terminal, generating two major isoforms, “short” and “long”, differing in 28 amino acids. NF-YC is spliced at the C-terminal, generating multiple isoforms, of which the 37kD and 50kD represent the most abundant, at the mRNA and protein levels. The splicings involve the Gln-rich trans-activation domains (TAD) present in both NF-YA and NF-YC, hence generating isoforms with identical HFD and DNA-binding properties, but potentially different activation potential [12,13]. These isoforms are expressed at various levels in different tissues and cell lines. 

NF-Y genes are generally not mutated nor amplified in human cancers, yet the trimer is known to activate many proliferative genes [14]. Indeed, no cell line has ever been shown to lack NF-Y activity, and transient functional inactivation of NF-YA leads to cell-cycle arrest and apoptosis in different cellular contexts. In addition, intersection of NF-Y genomic locations, as performed by the vast ENCODE consortium, found robust co-binding with oncogenic TFs, such as E2Fs, FOS, and MYC [15,16].

Compared to biochemical details of subunits interactions and DNA-binding, our knowledge on mRNA expression levels of NF-Y subunits in human tumors lags behind—higher NF-YA levels were reported in small cohorts of epithelial ovarian cancer [17,18], triple-negative breast cancers [19], and gastric cancer [20,21]. For this reason, we have recently started to investigate the TCGA RNA-Seq datasets—even a less than sophisticated view of the data found NF-YA overexpressed in tumors of epithelial origin [22]. We then decided to systematically analyze them—so far, we have completed analysis of breast carcinomas (BRCA) [22] and LUSC [23]. We report here on NF-Y subunit levels in lung adenocarcinomas by interrogating RNA-Seq datasets of TCGA and of two independent studies [24,25,26].

## 2. Materials and Methods

### 2.1. RNA-Seq Datasets

As of July 2019, there were RNA-Seq data on 576 LUAD samples [26], including 517 primary tumor samples and 59 non-tumor (normal) lung tissues. The data were downloaded from the http://firebrowse.org/ webpage. As for the GSE40419 and GSE81089 datasets, we retrieved the fastq files using the fastq-dump utility of the SRA toolkit 2.9.4 version. From the FASTQ files, we calculated mRNA expression with RSEM-1.3.1. The same procedure was used to obtain mRNA expression values of the 26 lung adenocarcinoma cell lines analyzed (DRP001919).

GSE81089 is a dataset of 198 NSCLC; using 42 genes validated classifier [27], we previously sub-classified samples in LUAD and LUSC [23]. This specifically identified 91 LUAD samples, which are analyzed in this study.

### 2.2. Classification of All TCGA LUAD Tumors

The current classification of LUAD in the three molecular subtypes made by TCGA referred to 230 tumors—we extended this to all LUAD tumors. To do this, the 506 genes previously validated as signatures for the three subtypes were used for this task [3]; each gene was median centered on all 517 LUAD samples and Pearson correlations were calculated between the predictor centroids and TCGA samples. A TCGA tumor’s subtype prediction was given by the centroid with the largest correlation value.

For the analyses involving LUAD cell lines, we calculated the median on each row (genes) of LUAD tumors, and we subtracted from the expression value the median calculated per gene (row). Then, we selected 166 upregulated genes in cell lines with low NF-YAs (“short”)/NF-YAl (“long”) ratio, used them as predictor centroid, and performed Pearson correlation to discriminate tumors with high and low ratio. 

### 2.3. Global Gene Expression Analysis

Differential gene expression (DEG) analysis of RNA-Seq data was performed using R package DESeq2 [28]. The tumor versus normal expression fold change (FC) denotes upregulation or downregulation according to the FC value. Log2FC, and the corresponding false discovery rate (FDR), were reported by the R package. FDR < 0.01 and |log2FC| > 2 were set as inclusion criteria for DEG selection in tumor/subtype versus normal samples. The same criteria were used to calculate DEG in cell lines with a low NF-YAs/NF-YAl ratio vs. high ratio, after the partition of cell lines in low (ratio < 1), intermediate (1 < ratio < 5), and high (ratio > 5). 

### 2.4. Gene Ontology, Pathway Enrichment, and Transcription Factor Binding Site Analysis

We used KOBAS 3.0 (http://kobas.cbi.pku.edu.cn/anno_iden.php) for pathway enrichment analysis using the ENTREZ gene IDs. The TFBS and de novo motif analysis were performed as in [22,23].

### 2.5. Analysis of Clinical Data

We retrieved clinical data related to the TCGA LUAD samples, including progression-free interval (PFI) time records of 345 patients, from the https://nborcherding.shinyapps.io/TRGAted/ web page [29]. Survival analysis was performed according to the Kaplan–Meier analysis and log-rank test [30]. Cox proportional hazard modeling of ratio and covariates was calculated to determine their independent impact on patient’s survival and to estimate the corresponding hazard ratio setting with the intermediate NF-YAs/NF-YAl ratio as the value of reference. 

### 2.6. Statistical Analysis

Analyses were performed in the R programming environment (version 3.2.5), with the ggplot2, ggsignif, reshape, ggpubr, clinfun, and heatmap.plus packages. Single comparisons between two groups were performed with the Wilcoxon rank sum test, whereas the Jonckheere’s Trend Test was used to verify significant trends between CIMP (CpG island methylation phenotype) and NF-YA global, NF-YAs, NF-YAl, and NF-YAs/NF-YAl ratios in a pre-ranked dataset.

## 3. Results

### 3.1. NF-YA Was Overexpressed in Lung Tumors

**Figure 1:**
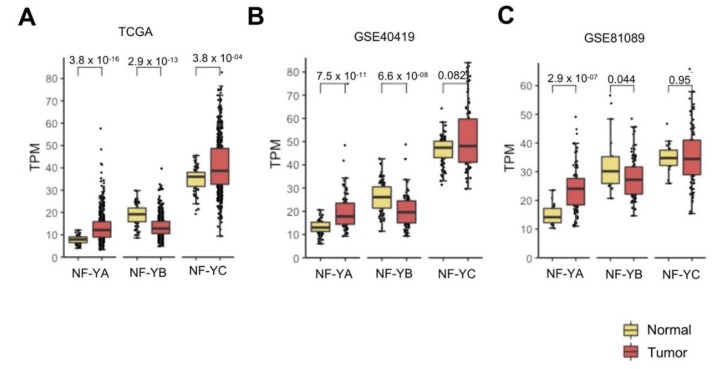
NF-YA was overexpressed in lung adenocarcinoma (LUAD). (**A**) Box plots of expression levels of the three NF-Y subunits at gene level in the TCGA LUAD cohort, measured in TPMs (Transcripts per Millions). (**B**) Same as (**A**), except that the GSE40419 dataset was analyzed. (**C**) Same as (**A**), except that 91 LUAD tumors were analyzed from the previously classified non-small cell lung carcinoma (NSCLC) GSE81089 dataset. *p*-values were calculated using a Wilcoxon signed-rank test.

We previously reported on NF-Y subunits in TCGA by Firebrowse (http://firebrowse.org/viewGene.html), indicating that NF-YA, not NF-YB/NF-YC, is elevated in epithelial tumors [22], including LUAD. We downloaded the entire set of 576 samples’ RNA-Seq dataset of TCGA [26] and confirmed that NF-YA levels were indeed increased in cancer compared to normal controls (*p*-value 10^−16^); instead, NF-YC had comparable levels and NF-YB was significantly (*p*-value 10^−13^) decreased (Figure 1A). The mRNA levels of HFD subunits were found to be globally higher than those of NF-YA in normal cells, but because of opposing changes, NF-YA and NF-YB levels were similar in tumors. 

Next, we interrogated two additional RNA-Seq datasets. The first, GSE40419, was derived from a Korean study of 89 LUAD [25]—mRNA levels of NF-YA were found to be substantially increased in tumors (*p*-value 10^−11^); NF-YB was decreased (*p* value 10^−8^) and NF-YC was unchanged (Figure 1B). The second study was derived from data on 198 NSCLC patients, mostly from Sweden [24], which we previously reclassified using signature genes in 78 LUSC and 91 LUAD [23]; in the current study, we focused on LUAD samples, and showed that NF-YA, but not the HFD subunits, was globally increased (Figure 1C). In conclusion, we confirmed NF-YA overexpression in LUAD, a decrease of NF-YB, and normal expression of NF-YC.

### 3.2. Splicing Isoforms of NF-YA Were Differentially Regulated in LUAD

**Figure 2:**
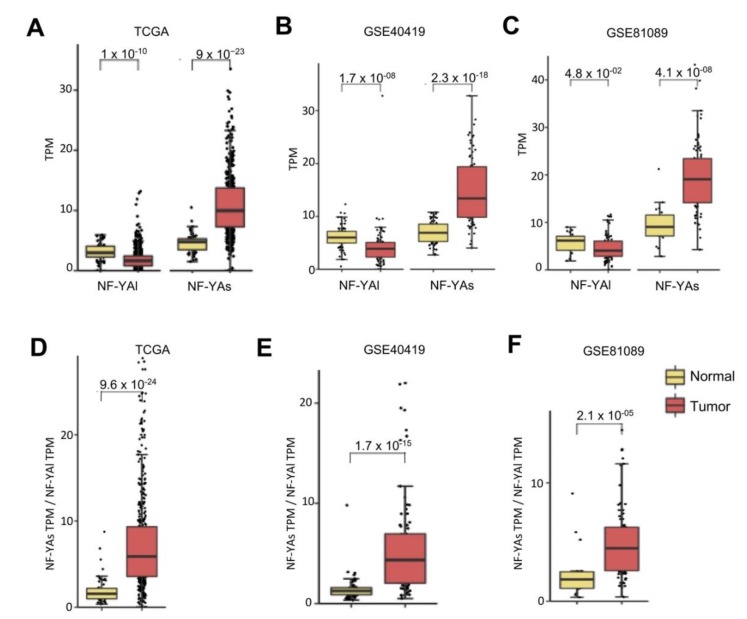
NF-YA short isoform was overexpressed in LUAD. (**A**) Box plots of expression levels of NF-YAs (“short”) and NF-YAl (“long”) in the TCGA LUAD dataset. (**B**) Same as (**A**), except that the GSE40419 dataset was analyzed. (**C**) Same as (**A**), except that LUAD tumors of the GSE81089 dataset were used. (**D**) Relative ratios of NF-YAs/NF-YAl in TCGA-LUAD. (**E**) Same as (**D**), except that the GSE40419 dataset was analyzed. (**F**) Same as (**D**), except that the LUAD tumors of the GSE81089 dataset were used.

We previously found a substantial change in NF-YA, but not NF-YC isoform expression in BRCA [22]. We analyzed the splicing isoforms in LUAD, first in the TCGA dataset. In normal cells, balanced levels of the two isoforms were found (Figure 2A); NF-YAs was increased (*p*-value 10^−23^), and NF-YAl decreased (*p*-value 10^−10^) in tumor samples. As for NF-YC, the predominant isoforms were the 37 kD (NF-YC2) and 50 kD (NF-YC1) (Figure S1)—their levels were essentially similar in normal and tumor samples. In the GSE40419 dataset, we observed a similar behavior (Figure 2B)—increase of NF-YAs (*p*-value 10^−18^) and decrease of NF-YAl (*p*-value 10^−8^). The GSE81089 data also showed a similar trend, less significant as to the NF-YAl decrease (Figure 2C). Again, NF-YC isoforms were overall not changed tumors (Figure S1). Because of the NF-YAs increase and NF-YAl drop, the NF-YAs/NF-YAl ratios were considerably increased in tumors (Figure 2D–F). Overall, these data concur that there was an isoform switch in NF-YA, but not NF-YC isoforms in LUAD, from normal to tumor cells. 

### 3.3. Expression of NF-YA Isoforms in LUAD Subtypes

According to gene expression, pathways profilings, and clinical features, LUAD tumors were classified in TRU (terminal respiratory unit, bronchioid), proximal-proliferative (PP, magnoid), and proximal-inflammatory (PI, squamoid)—the NF-YA overexpression could be restricted to one (or more) of the subtypes. TCGA has reported classification of 230 of the 517 tumors for which RNA-Seq data are currently available [26]. We first extended the subtype classification to all TCGA tumors. To do so, we used the gene signature as per Wilkerson et al. [3] and Girard et al. [27]. The new classification of all TCGA tumors is in Table S1. Figure S2A documents the robustness of our procedure. First, Venn diagrams show that the relative proportions were very similar—TRU were found to be 187, PP were 152, and PI 178. Second, a heatmap of a small nine-gene signature clearly partitioned the three subclasses (Figure S2B). Third, Figure S2C shows principal component analysis (PCA) of all TCGA samples, with robust clustering of the three subgroups. We did not attempt to further classify the GSE40419 and GSE81089 datasets, as the low numbers of individual subtypes would preclude obtaining statistically robust results.

**Figure 3:**
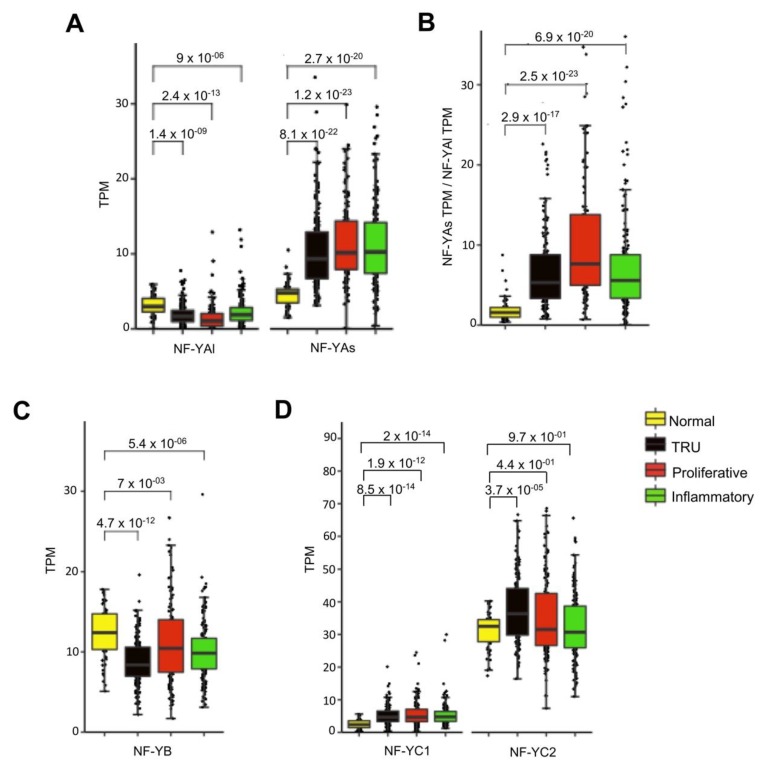
NF-YA was overexpressed in all LUAD subtypes. (**A**) Box plots represent the expression of NF-YA isoforms at gene level in LUAD subgroups, measured in TPM. (**B**) NF-YAs/NF-YAl ratios in the subgroups. (**C**) Same as (**A**), except that expression of NF-YB at gene level is shown. (**D**) Box plots of expression of NF-YC isoforms at gene level in LUAD subgroups. NF-YC1—50 kD isoform. NF-YC2—37 kD isoform. *p*-values were calculated using a Wilcoxon signed-rank test.

Having the complete list of TCGA LUAD subtypes, the levels of the three subunits were compared. A global increase of NF-YA, specifically NF-YAs, was observed in all subtypes (*p*-value < 2.7 × 10^−20/22/23^), whereas NF-YAl was significantly decreased (Figure 3A); consequently, the NF-YAs/NF-YAl ratios increased in all subtypes (Figure 3B). NF-YB was decreased, particularly in TRU (Figure 3C). As for NF-YC, there was a generalized increase in the less abundant NF-YC1 (50 kD) isoform, not so evident for the predominant 37 kD isoform (Figure 3D).

TCGA previously classified LUAD samples according to the global methylation of CpG islands (CIMP) in high (altered), intermediate, and low (normal) [26]—we stratified NF-YA expression (total, NF-YAs, NF-YAl, and NF-YAs/NF-YAl ratio) according to the CIMP status. Figure S3 shows the results—the CIMP^high^ cluster had slightly higher levels of NF-YA, with an opposite trend of NF-YAs (increased) and NF-YAl (decreased). Consequently, the NF-YAs/NF-YAl ratio increased in CIMP^high^ tumors.

In conclusion, overexpression of NF-YA was widespread and not restricted to specific subtype(s) of LUAD, and changes in HFD subunits were overall modest. NF-YAs^high^ tumors associated with an altered methylator phenotype.

### 3.4. LUAD Differentially Expressed Genes (DEG) had CCAAT in Promoters

**Figure 4:**
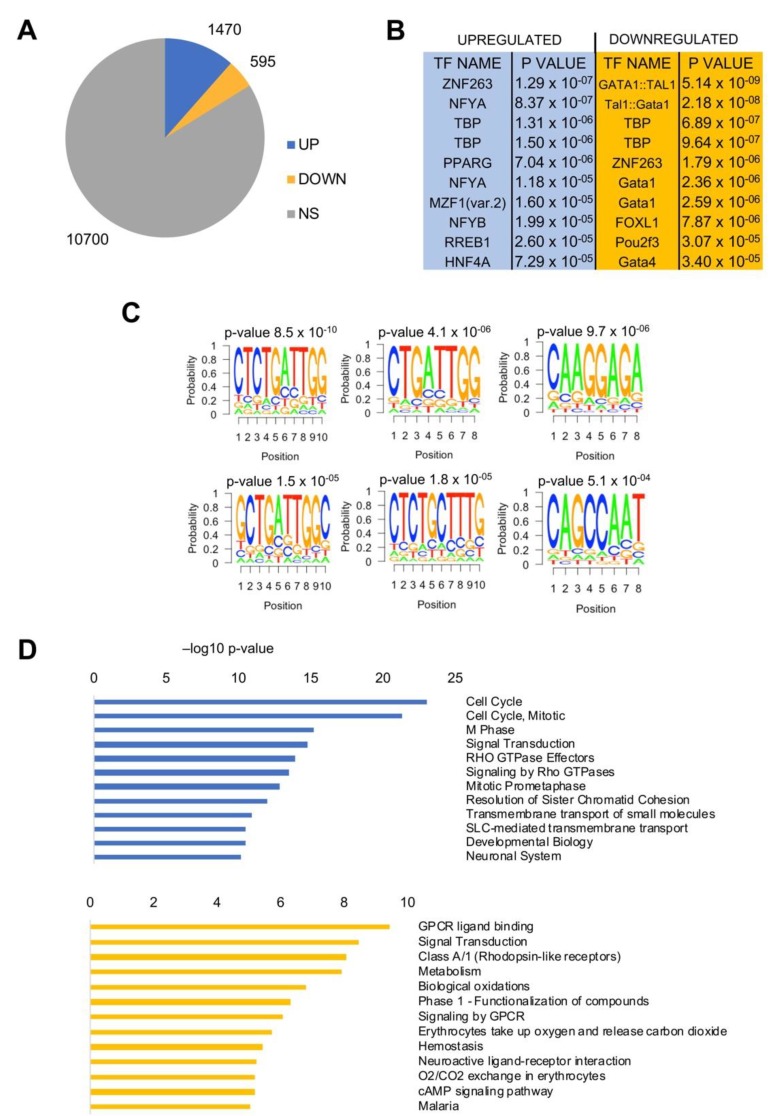
Gene expression analysis of LUAD TCGA tumors. (**A**) Up- and down-regulated genes in LUAD versus normal lung tissues. NS, not significant. (**B**) Pscan analysis of enriched TFBS in promoters (−450/+50 bps from the TSS) of up- and down-regulated genes in LUAD. (**C**) Weeder analysis of enriched de novo matrices in promoters of upregulated genes. (**D**) Reactome pathways enriched in upregulated genes (upper panel) and down-regulated genes (lower panel) listed according to their *p*-value. The list was obtained using KOBAS.

We compared RNA-Seq gene expression of all LUAD TCGA tumors to normal tissues. Using a Log2 FC > 2, *FDR* < 0.01 threshold, 1470 genes were found to be overexpressed and 595 were down-regulated (Figure 4A); the lists of all genes are found in Table S2. We then analyzed promoter sequences (−450 to +50 from the TSS) with the Pscan software [31], which identifies enriched DNA matrices present in the JASPAR database. The NF-Y matrix (NFYA/NFYB) was present in up-regulated genes, with robust *p*-values, but not in down-regulated genes, where other matrices—Gata, TBP, and FOX—predominate (Figure 4B). To further verify this finding, we ran another bioinformatic tool, Weeder, which captures matrices in promoters with no a *priori* bias [32]. The results are found in Figure 4C, showing that four of the top six matrices were related to CCAAT (or the reverse ATTGG). Finally, we used KOBAS to identify KEGG pathways in up- and down-regulated genes—in the former, *cell-cycle*, particularly related to the G2/M transition, came at the top of the list; in the latter, there was a more diversified set of terms (Figure 4D). *Signaling* was enriched in both DEGs, and thus was not very discriminatory. 

We then proceeded with a similar analysis of RNA-Seq of the three subtypes by using the threshold criteria used above. The lists of DEG genes are found in Table S2. CCAAT was absent in overexpressed genes in single subtypes. In pairwise matches, searches of TFBS found a robust enrichment of NF-Y sites only in the PI/PP overexpressed genes, together with E2Fs and Sp1/KLFs DNA motifs (Figure S4A). To confirm these findings, we ran Weeder on the PI/PP DEG cohort—Figure S4B shows retrieval of five CCAAT logos as the most enriched DNA sites. Finally, we analyzed pathways of overexpressed genes—Figure S4C shows that in the PI/PP commonly overexpressed cohort, the most statistically enriched categories were related to *cell-cycle*. 

We conclude that CCAAT was selectively present in genes overexpressed in LUAD at large, and in a cohort of 472 genes commonly up-regulated in the PI and PP cohorts, with an enrichment of proliferative cell-cycle genes.

Another interesting point to assess was the DEG of normal vs. tumor samples, partitioned according to the NF-YAs/NF-YAl ratio. Figure S5 shows that in tumors with a high NF-YAs/NF-YAl ratio (NF-YAs^high^), the NF-Y site was dominant in the promoters of the 1762 upregulated genes, and the prevalent KEGG terms were *cell-cycle* related. In the 1499 DEGs upregulated in tumors with a low NF-YAs/NF-YAl ratio (NF-YAl^high^) we also found cell cycle genes, but *extracellular matrix* terms were present. In this case, the NF-Y matrix dropped considerably in promoters of upregulated genes, and the most enriched motifs were of Zn finger TFs (Figure S5).

### 3.5. Analysis of DEG in LUAD Cell Lines and Tumors

**Figure 5:**
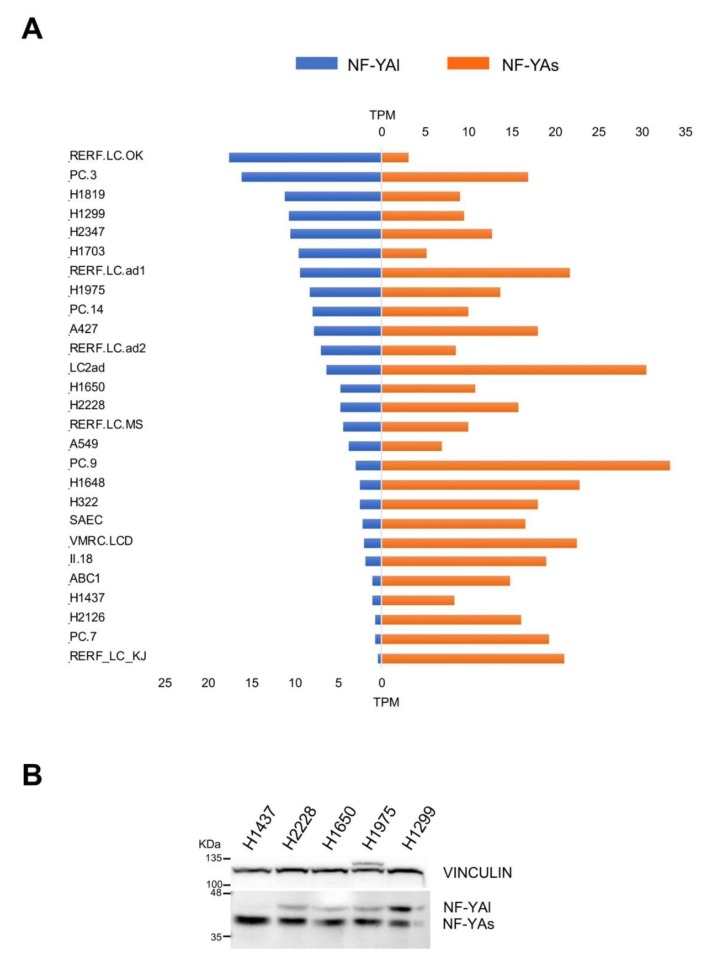
Expression levels of NF-YA isoforms in LUAD cell lines. (**A**) Relative levels of expression of the two NF-YA isoforms (TPMs) of the indicated LUAD cell lines as determined by RNA-Seq experiments. (**B**) Western blot analysis of NF-YA protein levels in the indicated representative LUAD cancer cell lines. Vinculin was used as an internal loading control.

RNA-Seq data were available from 26 lung adenocarcinoma and 1 normal-like cell lines. We analyzed these data, first to assess the NF-YAs and NF-YAl levels. A majority of cell lines, 16 in total, had large excess of NF-YAs, four—H1299, RERF.RC.OK, H1819, and H1703—had larger amounts of NF-YAl, and the rest had balanced levels, with a modest prevalence of NF-YAs (Figure 5A). We further checked the protein levels of the isoforms in selected cell lines. Figure 5B shows that H1437 had only NF-YAs, which paralleled the mRNA data (Figure 5A). At the opposite, H1299 had the highest amount of NF-YAl, whereas H2228, H1650, and H1975 had intermediate levels of the two isoforms with a prevalence of NF-YAs. In general, the mRNA and protein data were in very good agreement. 

**Figure 6:**
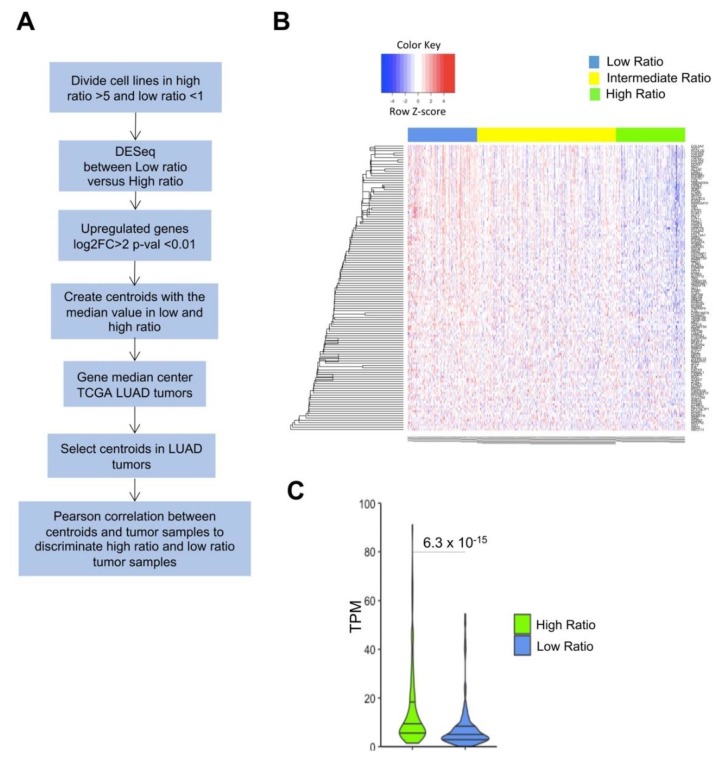
Identification and validation of a gene signature in NF-YAs/NF-YAl low ratio cell lines. (**A**) Overall scheme of the analysis. (**B**) Heatmap of expression levels of 125 genes identified as upregulated in low ratio cell lines. Color key indicates the *Z*-score. (**C**) Boxplots of NF-YAs/NF-YAl ratio distribution across TCGA LUAD samples after partitioning based on cell line signature. Analysis of the 130 centroid genes in LUAD tumors with high and low NF-YAs/NF-YAl ratios. *p*-values were calculated using a Wilcoxon signed-rank *Z*-test with Bonferroni correction.

To categorize BRCA and LUSC tumors, assessing the NF-YAs/NF-YAl ratio was more useful than the overall levels of the single subunits. We started a similar analysis with the LUAD cell lines dataset, and the workflow is depicted in Figure 6A. Figure S6 shows partitioning of lines according to high, intermediate, and low NF-YAs/NF-YAl ratios. Thereafter, we analyzed overexpressed genes in seven cell lines with low ratio, below one, that is, with relatively high levels of NF-YAl (Figure S6). We identified 166 genes commonly upregulated (FDR < 0.01 and log2 FC > 2), and 193 down-regulated. These DEGs were used as centroids to analyze LUAD TCGA tumors. The heatmap in Figure 6B shows a robust clustering of the 125 up-regulated cell lines related genes in the three tumors categories based on real NF-YAs/NF-YAl ratio value. We discarded 41 genes whose median values were similar in tumor tissues; in addition, significant Wilcoxon rank test (*p*-value 10^−15^) of mean expression within tumors having a low NF-YAs/NF-YAl ratio is shown in Figure 6C. 

Reassured that this cell line-derived centroid was predictive of isoform ratio in tumors, we performed KOBAS analysis on the up-regulated genes, finding terms such as *collagen degradation*, *extracellular matrix*, and *integrin* as reflective of a loss of epithelial features. As a control, we ran KOBAS on down-regulated genes in low ratio cell lines, with terms such as *metabolism* and *signal transduction*, prominent in the pro-proliferative signature of high ratio tumors with relatively high NF-YAs, being found in this cluster (Figure S7). In summary, we can conclude that NF-YAs^high^ cell lines and tumors had a proliferative signature, whereas higher levels of NF-YAl were associated with a different signature.

### 3.6. LUAD Subclasses Had Different NF-YAs/NF-YAl Ratios

**Figure 7:**
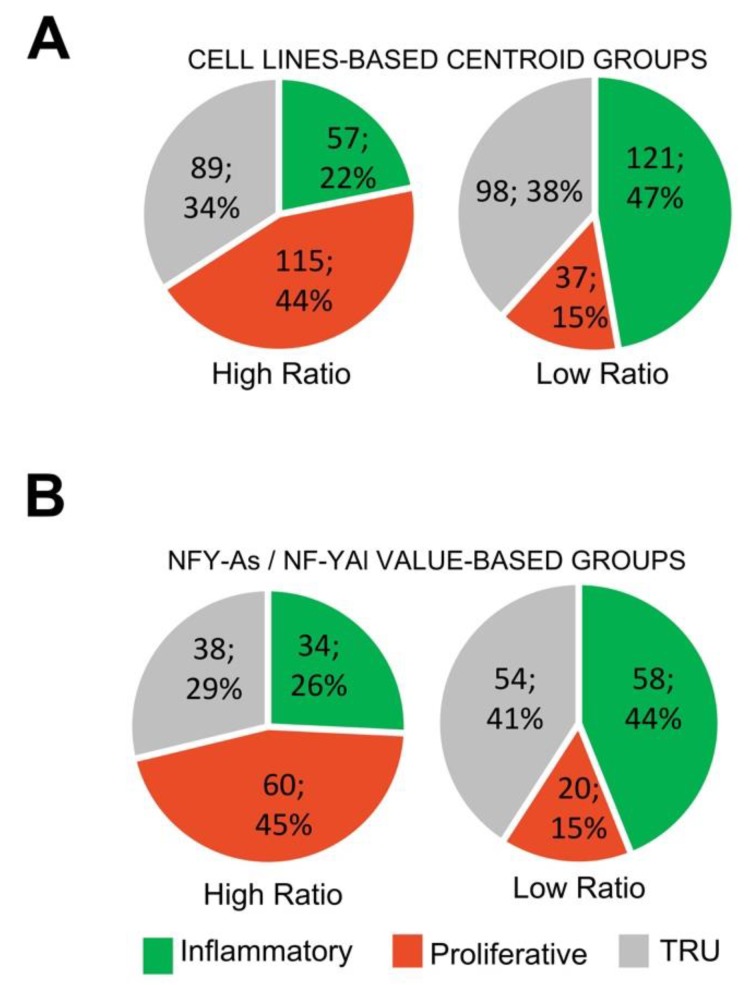
LUAD subclasses have different NF-YAs/NF-YAl ratios. (**A**) Subgroup distribution of LUAD tumors partitioned in high (left) and low (right) NF-YAs/NF-YAl ratios, according to the cell line-based centroids. (**B**) Subgroup distribution of LUAD tumors partitioned on the basis of sample NF-YAs/NF-YAl ratios.

We wondered whether there would be any skewing in LUAD subtypes on the basis of partitioning of high vs. low NF-YAs/NF-YAl ratios. To assess this, we took two approaches. First, we considered the cell line gene expression centroid used above to partition tumors arbitrarily into high and low ratios. Figure 7A shows that in tumors predicted to be high ratio, on the basis of DEGs, the majority (44%) were of the PP sub-type, and PI was only 22%. The opposite happened with those predicted to be low ratio, 47% were PI and only 15% were PP. To verify this, we stratified all TCGA tumors on the basis of the actual NF-YAs/NF-YAl ratio, monitoring the partition between the three subclasses. Figure 7B shows that in PP tumors, the vast majority (45%) were high ratio, and, conversely, in the PI subclass, most (44%) were low ratio. As for TRU tumors, they showed a similar distribution of high and low NF-YAs/NF-YAl ratios. In conclusion, these data concurred that the centroid identified in cell lines was also predictive of NF-YAs/NF-YAl ratio in subclasses, and indicated that there was indeed skewing, specifically in PI vs. PP tumors.

### 3.7. Clinical Outcomes of NF-YA Isoform Ratios in LUAD

**Figure 8:**
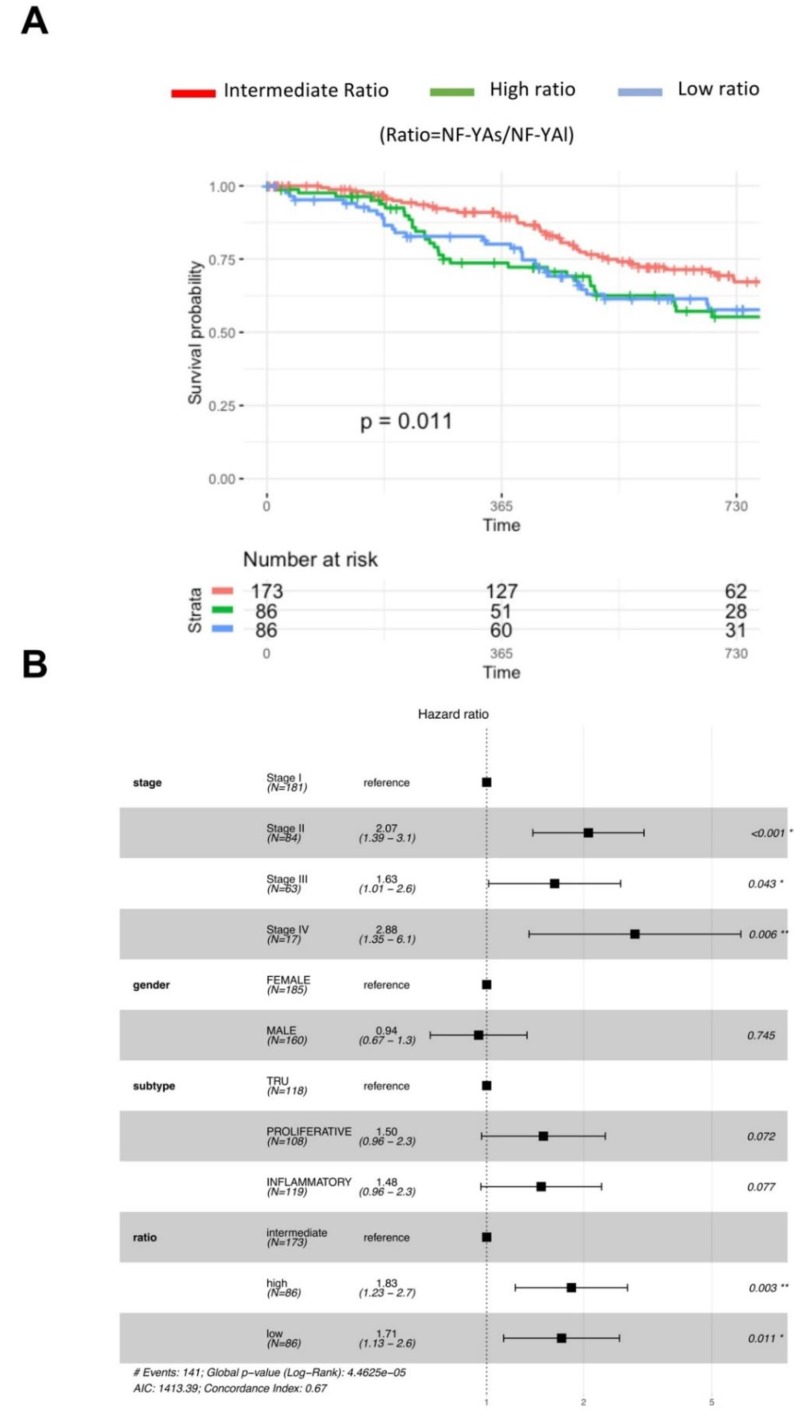
Clinical outcome of LUAD tumors with different NF-YAs/NF-YAl ratios. (**A**) Progression-free interval curves of survival probability of LUAD tumors with stratification according to quartiles of NF-YAs/NF-YAl ratios (intermediate, high, and low). (**B**) Hazard ratios of the three cohorts as above, according to stage (I–IV), gender, sub-type (Basal set as reference), and NF-YAs/NF-YAl ratios. *p*-values were calculated using a Cox proportional hazards regression analysis (See Section 2.5).

We next considered clinical data of tumors with low and high NF-YAs/NF-YAl ratios. We correlated ratios with progression-free intervals (PFI) [30], by considering all tumors for which clinical features were available (345 in total) partitioned into three cohorts: first quartile with NF-YAs^high^, last quartile with NF-YAl^high^, and the two middle quartiles with intermediate levels of the isoforms. The curves of Figure 8A show a drop in PFI in patients with either high or low ratios, compared to those with intermediate ratios (*p*-value 0.024).

LUAD has four clinical stages (I–IV). Figure 8B shows hazard ratios increasing significantly already at stage II; no effect on gender nor subclass (PI set as standard) was scored. Instead, setting intermediate NF-YAs/NF-YAl ratio as a reference, we reported a statistically increased hazard both in high (1.83, *p*-value 0.003) and in low ratio tumors (1.71, *p*-value 0.011). We conclude that the clinical outcome of LUAD was impacted by the relative levels of the NF-YA isoforms, and a worse prognosis was observed in patients with either high or low NF-YAs/NF-YAl ratios.

## 4. Discussion

Alteration of the expression of NF-YA in tumors has only very recently received attention, including from our lab. As the third chapter of our systematic interrogation of NF-Y expression in human tumors, we have provided analysis of LUAD in TCGA and two independent RNA-Seq datasets of patients with different ethnic backgrounds, as well as cell lines. The relative expression levels of the two major NF-YA isoforms, whose logic has been missing for decades, is becoming clearer, at least in tumors of epithelial origin. The data confirm and extend to LUAD three types of results: the high frequency of CCAAT boxes in promoters of genes globally overexpressed in cancer; the increased expression of the NF-YA “short” subunit in most, but not all tumors and concomitant decrease of NF-YAl; the worse clinical outcome of tumors with high, as well as low NF-YAs/NF-YAl ratios.

CCAAT boxes abundance in promoters of genes overexpressed in different types of cancers was shown a decade ago in microarray profiling experiments [5]. This set of reports culminated in a vast Oncomine microarray analysis, finding CCAAT as one of the most represented matrices [10]. Recent analysis of quantitatively more precise sets of RNA-Seq data confirm this [8,9]. We have started to tackle this issue systematically, analyzing breast carcinomas (BRCA) and lung squamous cell carcinomas (LUSC), finding that promoters of overexpressed DEGs are indeed CCAAT-enriched. As a corollary, these CCAAT genes are pro-proliferative, centered on cell-cycle and signaling GO terms [22,23]. LUAD data confirm this, with one notable difference: although CCAAT was enriched in the core sets of overexpressed DEG common to all molecular subtypes of BRCA and LUSC, in LUAD, only the PP/PI intersection had the typical DEG profile with CCAAT and *cell-cycle* terms; TRU tumors lacked it.

A few points can be made about these findings. First, CCAAT has yet to be found in promoters of genes down-regulated in cancer. Our data on down-regulated DEG in LUAD are yet another confirmation of this. Because CCAAT is a relatively frequent element of human promoters, found in metabolic, inducible, tissue-specific genes as well, this dichotomy is indicative of systematic differences in promoter “structure” of DEG genes. Second, NF-Y is essential for activation of many cell-cycle genes, particularly all G2/M genes, a hallmark of the “proliferative” signatures often detected in cancers [5]. Third, as in BRCA and LUSC, CCAAT is joined by selected TFBSs belonging to the E2Fs and GC-rich galaxies. Robust evidence of colocalization, as well as functional interplay on selected promoters, have been reported for E2F1/4 and Sp1/Sp2 [5,14,33,34,35]. In particular, the role of overexpressed E2F family members in lung cancer is well documented [36,37]. It is becoming clear that a much closer structural attention must be paid to the fine structure of this class of promoters in order to eventually understand TF synergistic themes.

A further notable difference with respect to BRCA and LUSC is that NF-YB was found to be less expressed in LUAD with respect to normal samples, with the number of mRNA molecules approaching that of NF-YA, which is usually the least expressed of the subunits at the mRNA level. Thus, the excess of NF-YC mRNAs, already reported in BRCA and LUSC, was found to be even more pronounced. This expression dichotomy needs to be understood from the protein standpoint, as HFDs are apparently obligate heterodimers, very much like core histones H2A/H2B or H3/H4. This is even more so because NF-YA and NF-YB are nuclear proteins, but NF-YC is also found in the cytoplasm, including after overexpression of Flag- and GFP-tagged proteins. Indeed, it was shown as becoming stably nuclear only through a piggy-back mechanism mediated by NF-YB association [38,39]. Hence, a decrease of NF-YB expression in LUAD, irrespective of an increase of NF-YC in tumors, might entail a decrease of nuclear HFD subunits. Why is more NF-YA expressed in tumors if the nuclear levels of NF-YB, and hence the HFDs, become lower and potentially limiting? Obviously, control of protein levels via post-transcriptional mechanisms might alleviate and balance this apparent paradox. For example, the half-life of the NF-YA proteins was calculated in 1–2 h, much shorter than that of NF-YB. All these aspects, escaping the present analysis, deserve further consideration.

The third difference with respect to our previous analysis concerns the population of tumors having low NF-YAs/NF-YAl ratios. We recently showed that a BRCA NF-YAl^high^ population is specifically restricted to Claudin^low^ tumors, a portion of the Basal-like subtype; a cohort of tumors with these features also exists in LUAD but, similarly to LUSC, they do not belong to any of the molecularly characterized tumor subtypes.

The two major NF-YA splicing isoforms differ in the Gln-rich trans-activation domain (TAD), with NF-YAl harboring 28/29 extra aminoacids coded by Exon 3. The C-terminal subunits-interaction and DNA-binding parts are shared by NF-YAs and NF-YAl, leading to similar trimerization and CCAAT-binding of the two isoforms. Thus, differential splicing is predicted to affect activation potential, and data supporting this were reported in mESCs (Mouse Embryonic Stem Cells) [40]. The mechanistic details, for the time being, are completely unknown. 

Our gene expression profiles in LUAD cell lines identified DEG up-regulated in NF-YAl^high^ cells, which were found to be mostly down-regulated in NF-YAs^high^ cells. This signature was used to characterize TCGA tumors with a low NF-YAs/NF-YAl ratio—we confirmed that this pattern can be observed in a discrete cohort of LUAD tumors in vivo. Remarkably, the PFI curves were similar to those with NF-YAs^high^, namely, worse than those of patients with intermediate isoforms levels. We showed that this group of genes had two important features: a pro-migration (extracellular matrix/collagen) signature and the absence of CCAAT in the promoters. The first was visibly different from the proliferative signature associated to the bulk of NF-YAs^high^ tumors. The second signaled that NF-YAl activity is very likely exerted indirectly—not through CCAAT-binding and direct activation of all genes, but on very selected TF regulators, yet to be identified. 

## 5. Conclusions

Tumors with intermediate NF-YAs/NF-YAl ratios were found to have better prognosis, suggesting that the balance between the two subunits is important. High amounts of NF-YAl, compared to NF-YAs, drove a program of overexpression of mesenchymal genes, not targeted by NF-YAs. We can hypothesize that lower amounts of NF-YAl could be unable to do that, while competing with NF-YAs on pro-growth, cell cycle promoters, with a different functional outcome. Similarly, lower amounts of NF-YAs would be less efficient in activating these genes. Clearly, all this needs to be verified at the single cell level, a task that is important to perform in the future to clarify these issues. Note that we reported an identical scenario in LUSC and a similar scenario in BRCA, but specific only to Basal-like tumors; thus, a trend is becoming apparent, at least in epithelial tumors. The identification of the NF-YAl-targeted TFs promoting expression of mesenchymal genes should also be considered a priority.

## Supplementary Materials

The following are available online at https://www.mdpi.com/2073-4425/11/2/198/s1. Figure S1: Splicings of NF-YC. (A) Box plots represent the expression levels of NF-YC isoforms across TCGA dataset. (B) NF-YC splicing isoforms in GSE404119. (C) NF-YC1 and NF-YC2 in GSE81089. NF-YC1 isoform encodes for the 50 kD protein whereas NF-YC2 encodes for 37 kD. Figure S2: Partitioning of LUAD TCGA tumors in molecular subtypes. (A) Venn diagrams of the three LUAD subtypes—smaller circles represent previous TCGA classification of 230 tumors and larger circles represent complete classification of all LUAD tumors. (B) Heatmap of LUAD samples using nine signature genes, on the basis of the molecular classification [3]. (C) PCA (principal component analysis) of the LUAD subtype samples classified according to the gene signature described by Girard et al. Figure S3: NF-YA expression in CpG island methylation phenotypes. (A) Box plots showing the expression levels of NF-YA in CpG island methylator phenotype (CIMP)-L, CIMP-I, and CIMP-H. (B) Same as (A), but with NF-YAs expression. (C) Levels of NF-YAl in the three methylation status classes. (D) NF-YAs/NF-YAl ratio in low, intermediate, and high CpG island methylated tumors. Figure S4: Gene expression analysis of the three LUAD subgroups. (A) Venn diagram of the genes overexpressed in the four subgroups of LUAD. Pscan analysis of the TFBS enriched in the 472 overexpressed genes, both in proliferative and inflammatory subtypes. (B) Weeder analysis of the novo motif identification of sites enriched in promoters of the 472 genes over-expressed in inflammatory and proliferative subtypes. *p*-values were calculated using a Wilcoxon signed-rank *Z*-test with Bonferroni correction. (C) KEGG terms enriched in the same 472 upregulated genes. Figure S5: KEGG-enriched terms in LUAD high or low NF-YAs/NF-YAl ratio tumors vs. normal samples. (A) KEGG-enriched terms in LUAD high NF-YAs/NF-YAl ratio tumors vs. normal sample upregulated genes (left panel) and Pscan analysis of the TFBS enriched in the in overexpressed genes (right panel). (B) KEGG-enriched terms in LUAD low NF-YAs/NF-YAl ratio tumors vs. normal sample upregulated genes (left panel) and Pscan analysis of the TFBS enriched in the in overexpressed genes (right panel). Figure S6: Table of lung adenocarcinoma cell lines. List of LUAD cell lines names, NF-YAs/NF-YAl ratio values, and ratio classes: low (ratio < 1), intermediate (1 < ratio < 5), and high (ratio > 5). Figure S7: KEGG-enriched terms in LUAD low vs. high NF-YAs/NF-YAl ratio cell lines. Pathways enriched in up-regulated genes (upper panel) and down-regulated genes (lower panel) of low compared to high ratio cell lines.
