# Supplementary material for: NF-YA Overexpression in Lung Cancer: LUAD
Source: Genes (Basel). 2020 Feb 14;11(2):198. doi: 10.3390/genes11020198 (PMC7074112; doi:10.3390/genes11020198)

Supplementary Figure 1

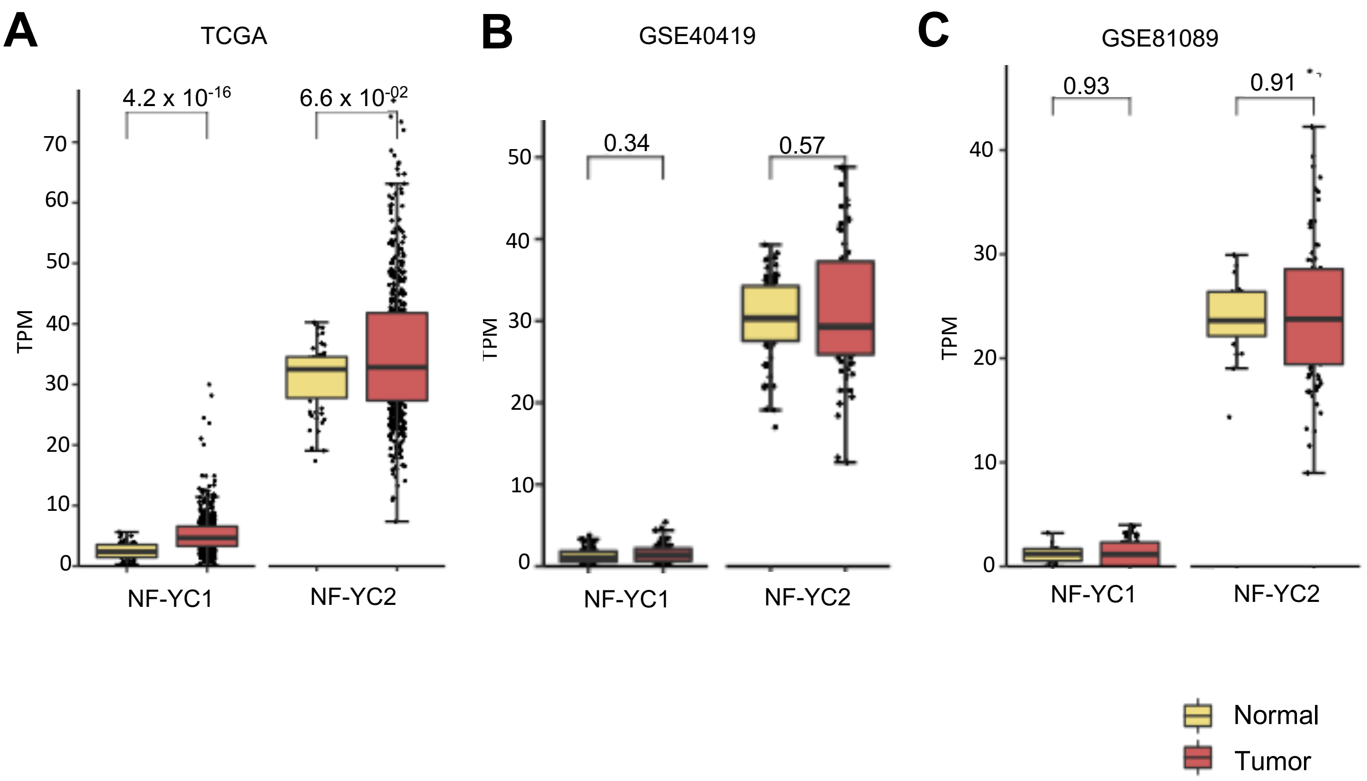

Supplementary Figure 2

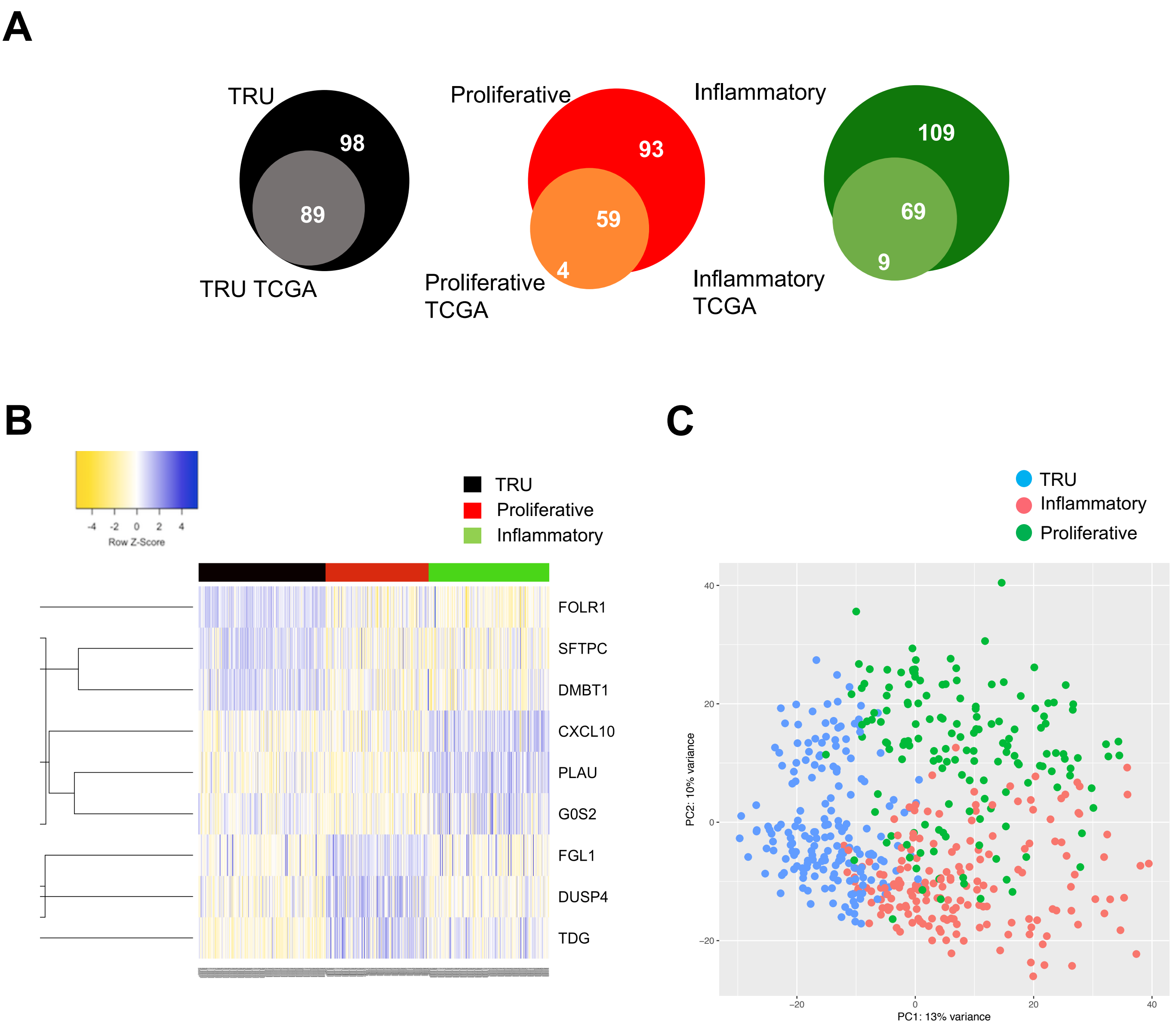

Supplementary Figure 3

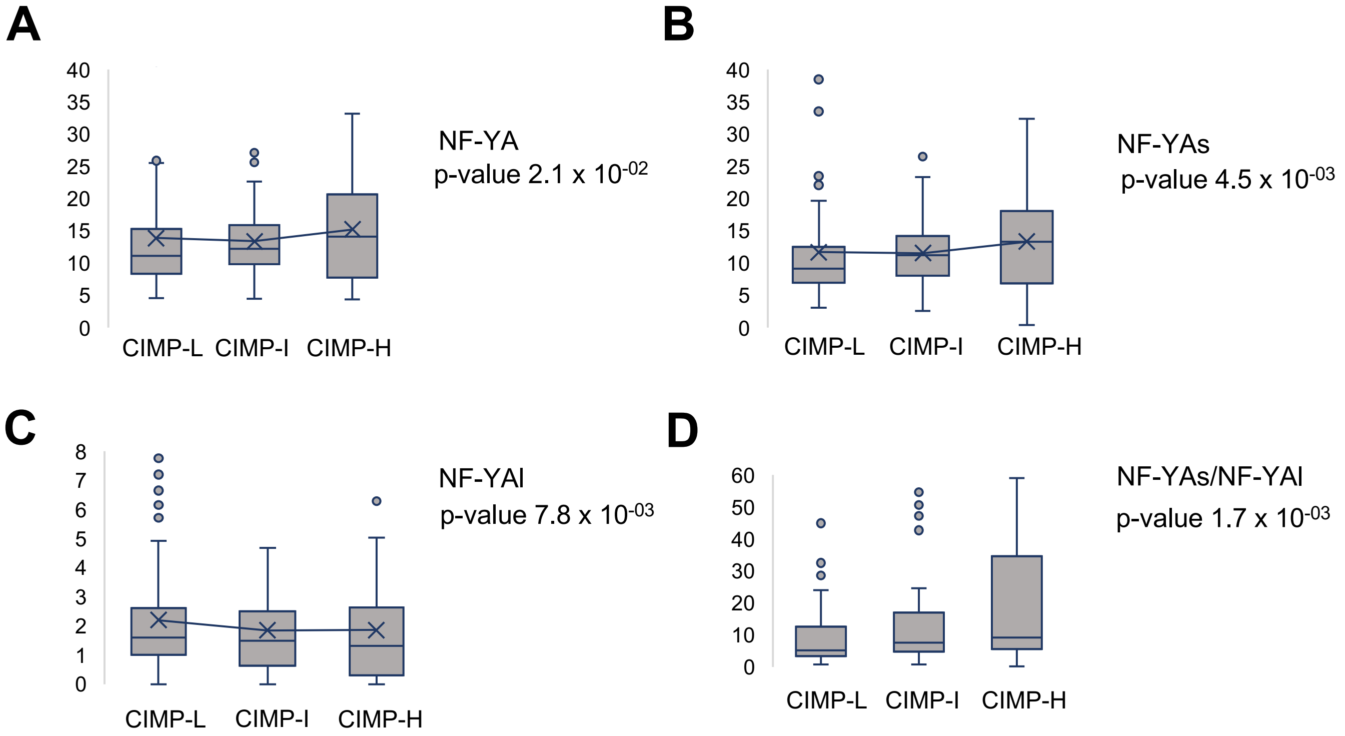

CIMP-L ->Low Methylation
CIMP-I ->Intermediate Methylation
CIMP-H->High Methylation

Supplementary Figure 4

A

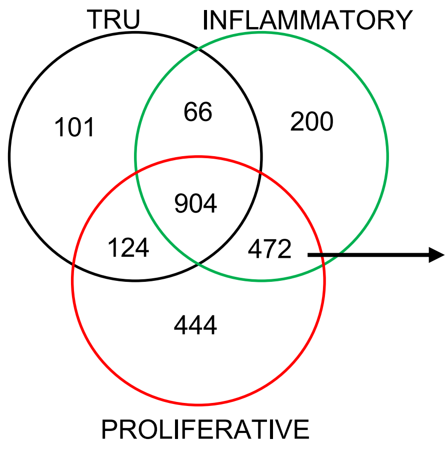

TF NAME	P VALUE
NFYA	3.13×10^{-17}
NFYA	3.24×10^{-16}
NFYB	2.46×10^{-15}
E2F4	3.05×10^{-13}
E2F1	6.75×10^{-10}
E2F1	9.75×10^{-10}
E2F3	1.51×10^{-08}
E2F6	8.63×10^{-08}
E2F8	3.22×10^{-07}
Gmeb1	8.79×10^{-06}
E2F7	1.28×10^{-05}
NRF1	3.55×10^{-05}
ZIC3	5.57×10^{-05}
EGR2	6.28×10^{-05}
Gfi1	7.11×10^{-05}
ZBTB33	8.07×10^{-05}

B

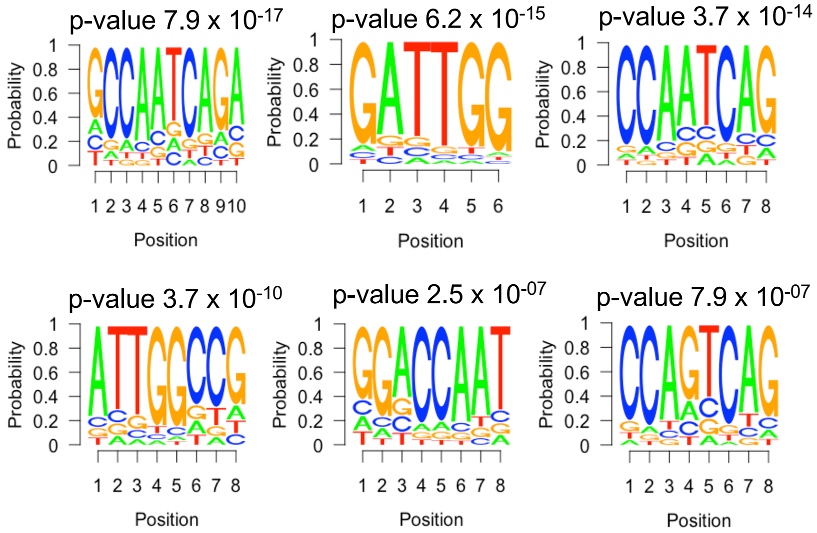

C

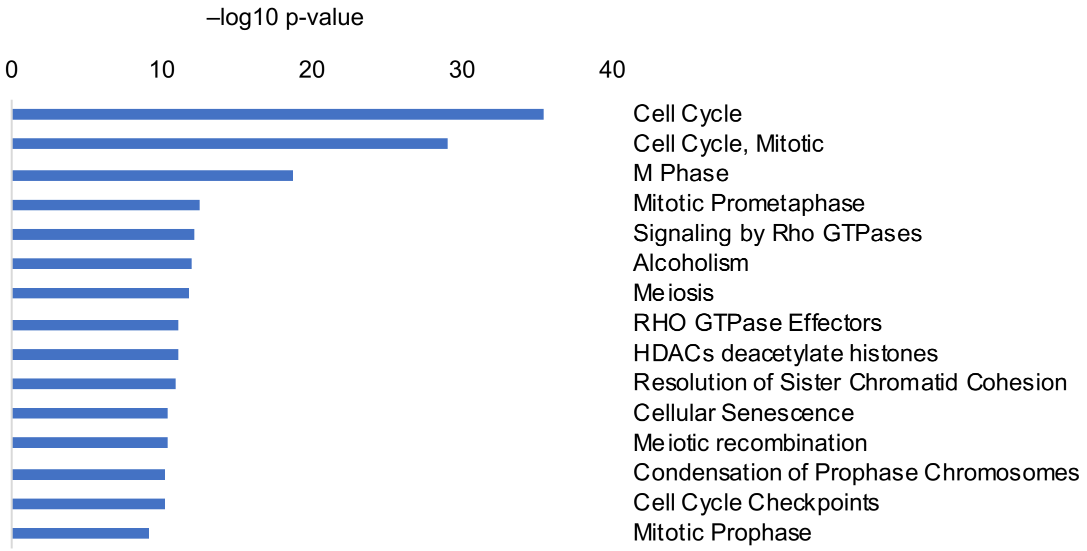

Supplementary Figure 5

A

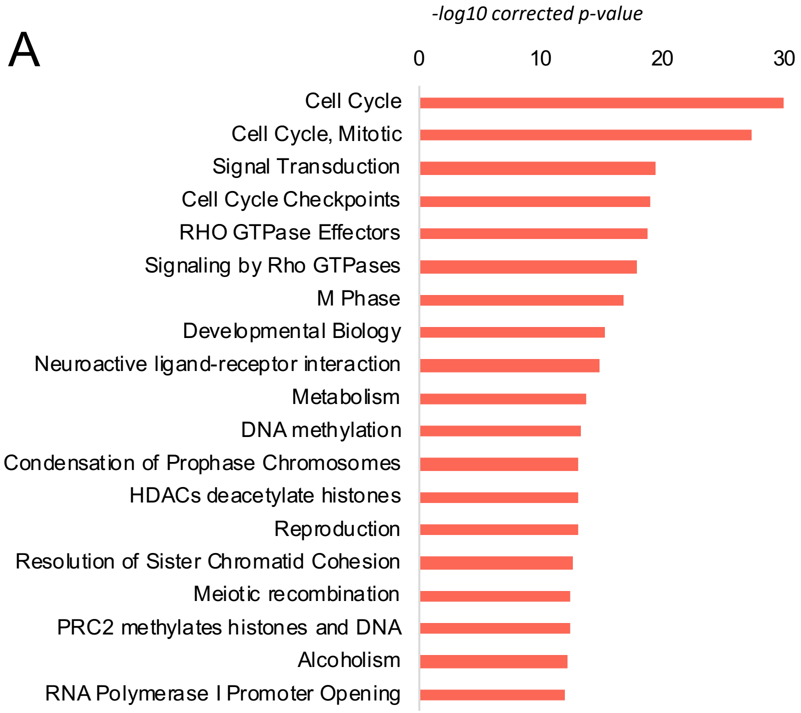

TF NAME	P VALUE
NFYA	9.27 x 10^{-08}
NFYA	2.17 x 10^{-07}
ZNF263	2.97 x 10^{-07}
E2F6	7.28 x 10^{-07}
ID4	1.68 x 10^{-06}
TCF4	1.75 x 10^{-06}
HNF4A	2.06 x 10^{-06}
ZNF740	2.12 x 10^{-06}
Pparg::Rxra	2.37 x 10^{-06}
TCF3	2.84 x 10^{-06}
ESR1	4.62 x 10^{-06}
ESR2	5.45 x 10^{-06}
NFYB	5.88 x 10^{-06}
E2F4	7.24 x 10^{-06}
NR2C2	1.03 x 10^{-05}
REST	1.49 x 10^{-05}
RREB1	2.23 x 10^{-05}
ZIC3	2.27 x 10^{-05}
ZIC1	2.67 x 10^{-05}
FIGLA	2.72 x 10^{-05}
ZEB1	3.30 x 10^{-05}
Bach1::Mafk	3.39 x 10^{-05}

B

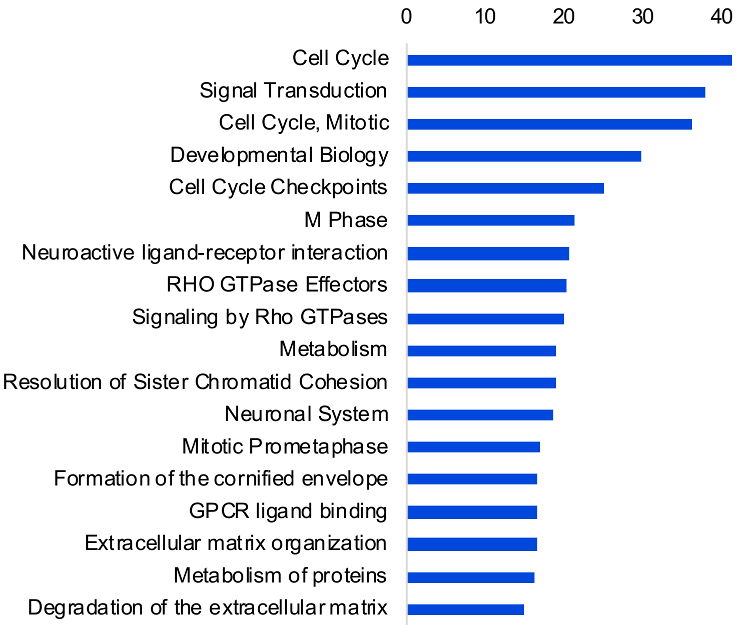

TF NAME	P VALUE
ZNF263	1.85 x 10^{-09}
ZNF740	1.12 x 10^{-08}
RREB1	5.84 x 10^{-08}
E2F6	5.97 x 10^{-08}
FOSL1	1.73 x 10^{-06}
TCF4	2.30 x 10^{-06}
ID4	2.78 x 10^{-06}
Bach1::Mafk	3.40 x 10^{-06}
MZF1(var.2)	4.02 x 10^{-06}
JUN(var.2)	6.84 x 10^{-06}
KLF5	7.44 x 10^{-06}
MZF1	8.38 x 10^{-06}
EWSR1-FLI1	9.31 x 10^{-06}
JUNB	9.58 x 10^{-06}
Tcf3	1.26 x 10^{-05}
TCF3	1.39 x 10^{-05}
EGR2	1.73 x 10^{-05}
E2F4	2.78 x 10^{-05}
TBP	3.23 x 10^{-05}
TBP	3.24 x 10^{-05}
EGR4	3.76 x 10^{-05}
NFYA	4.23 x 10^{-05}

Supplementary Figure 6

Cell line	Ratio	Classification
RERF_LC_OK	0.13676633	Low ratio
H1703	0.50457782	Low ratio
H1299	0.64939271	Low ratio
H1819	0.66888519	Low ratio
PC3	0.84865791	Low ratio
H2347	0.90432612	Low ratio
RERF_LC_ad2	0.92317224	Low ratio
PC14	1.07947805	Medium ratio
A549	1.29844098	Medium ratio
H1975	1.42295082	Medium ratio
A427	1.51476378	Medium ratio
H1650	1.58732877	Medium ratio
RERF_LC_MS	1.68342644	Medium ratio
RERF_LC_ad1	1.7962298	Medium ratio
H2228	2.36138614	Medium ratio
LC2_ad	4.12834979	Medium ratio
H1437	5.41216216	High ratio
H322	5.97916667	High ratio
H1648	6.69817073	High ratio
IL18	7.08661417	High ratio
VMRC_LCD	7.59233449	High ratio
PC9	9.68674699	High ratio
ABC1	10.5367647	High ratio
H2126	14.3394495	High ratio
PC7	20.1182796	High ratio
RERF_LC_KJ	38.5454545	High ratio

Supplementary Figure 7

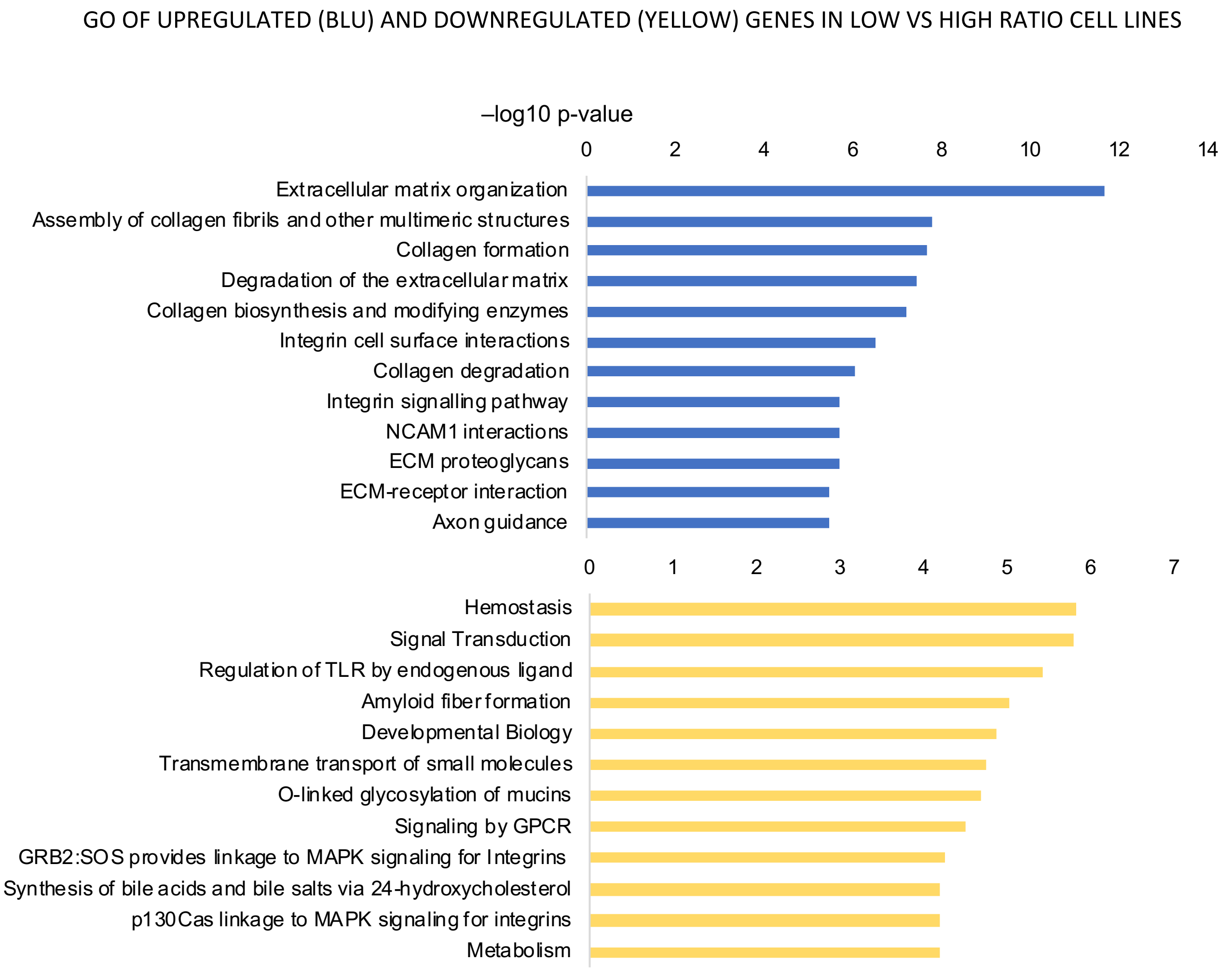

Supplement: Supplementary file 1 [file genes-11-00198-s001.zip › Supplementary/SupplLUADrev.pdf]
